# Dopaminergic neuron injury in Parkinson’s disease is mitigated by interfering lncRNA SNHG14 expression to regulate the miR-133b/ α-synuclein pathway

**DOI:** 10.18632/aging.102330

**Published:** 2019-11-04

**Authors:** Li-Min Zhang, Meng-Han Wang, He-Cheng Yang, Tian Tian, Gui-Fang Sun, Yang-Fei Ji, Wen-Tao Hu, Xi Liu, Jian-Ping Wang, Hong Lu

**Affiliations:** 1 Department of Neurology, The First Affiliated Hospital of Zhengzhou University, Zhengzhou, Henan 450052, China

**Keywords:** Parkinson’s disease, SNHG14, miR-133b, α-synuclein, dopaminergic neuron injury

## Abstract

This study explored the influence of long non-coding RNA (lncRNA) SNHG14 on α-synuclein (α-syn) expression and Parkinson’s disease (PD) pathogenesis. Firstly, we found that the expression level of SNHG14 was elevated in brain tissues of PD mice. In MN9D cells, the rotenone treatment (1μmol/L) enhanced the binding between transcriptional factor SP-1 and SNHG14 promoter, thus promoting SNHG14 expression. Interference of SNHG14 ameliorated the DA neuron injury induced by rotenone. Next, we found an interaction between SNHG14 and miR-133b. Further study showed that miR-133b down-regulated α-syn expression by targeting its 3’-UTR of mRNA and SNHG14 could reverse the negative effect of miR-133b on α-syn expression. Interference of SNHG14 reduced rotenone-induced DA neuron damage through miR-133b in MN9D cells and α-syn was responsible for the protective effect of miR-133b. Similarly, interference of SNHG14 mitigated neuron injury in PD mouse model. All in all, silence of SNHG14 mitigates dopaminergic neuron injury by down-regulating α-syn via targeting miR-133b, which contributes to improving PD.

## INTRODUCTION

Parkinson’s disease (PD) is the second most common progressive neurodegenerative disease characterized by the aggregation of α-synuclein (α-syn) neuronal inclusions, and a massive loss of dopaminergic (DA) neurons [1, 2]. Rotenone is a widely used insecticide that causes parkinsonian features such as loss of DA neurons, therefore PD experimental models were developed by utilization of rotenone to reveal the neurodegenerative mechanism in PD [3].

Previously, it has been revealed that point mutations in the α-syn gene (SNCA) is a rare cause of familial PD and α-syn protein is a component of Lewy bodies and Lewy neurites from idiopathic PD [4]. The aggregation of α-syn protein is the pathological hallmark of PD [5, 6], exhibiting that α-syn has the potential to be a diagnostic biomarker of PD [7]. There is an evidence for the occurrence of dopamine neuron programmed death in several neurodegenerative disorders including PD [8]. In PD, the main form of DA neurons programmed death is apoptosis [9], which could be regulated by α-syn [10]. Recently, it has been reported that the inhibition of α-syn contributes to ameliorating the arsenite-induced apoptotic cell death in the DA PC12 cells [11], and rotenone induced an up-regulation of α-syn in human DA neuroblastoma cells [12], validating the significance of α-syn expression in DA neurons and its impact on PD.

MicroRNA-133b has been identified to be specifically expressed in midbrain DA neurons and to be deficient in midbrain tissue from patients with PD [13]; and serum miR-133b expression levels were significantly decreased in PD patients [14]. Moreover, ameliorative effect of miR-133b on axon degeneration induced by neurotoxin MPP+ has been discussed [15]. These findings powerfully proved that miR-133b is closely related to the development of PD. The complementary binding sites between miR-133b and the 3′-UTR of the gene encodes α-syn, SNCA, were predicted with bioinformatics analysis, implying the potential interaction between them in DA neurons.

Synphilin-1 (SP-1) is a key transcription factor, which was interacted with α-syn and has implications in PD pathogenesis [16]. In PC12 cells treated with MPP+, the expression of SP-1 mRNA and protein were increased [17]. With bioinformatics analysis and luciferase reporter assays, Liu et al. revealed that SP-1 could bind to the promoter region of long noncoding RNA (lncRNA) SNHG14, leading to the overexpression of SNHG14 [18]. Furthermore, SNHG14 overexpression significantly promoted microglia activation in cerebral infraction [19]. In our preliminary study, clear up-regulation of SNHG14 was noted after rotenone treatment, and potential binding sites between SNHG14 and miR-133b were forecast. Taken together, we speculated that rotenone may up-regulate SNHG14 through SP-1, which contributes to the inhibition of miR-133b and accumulation of α-syn, and thus aggravating neuron injury in PD. Therefore, this study was undertaken to validate this hypothesis, aiming to explore the etiology and pathogenesis of PD and provide scientific evidence for the prevention and treatment of this neurodegenerative disorder.

## RESULTS

### SNHG14 expression was increased in PD

**Figure 1:**
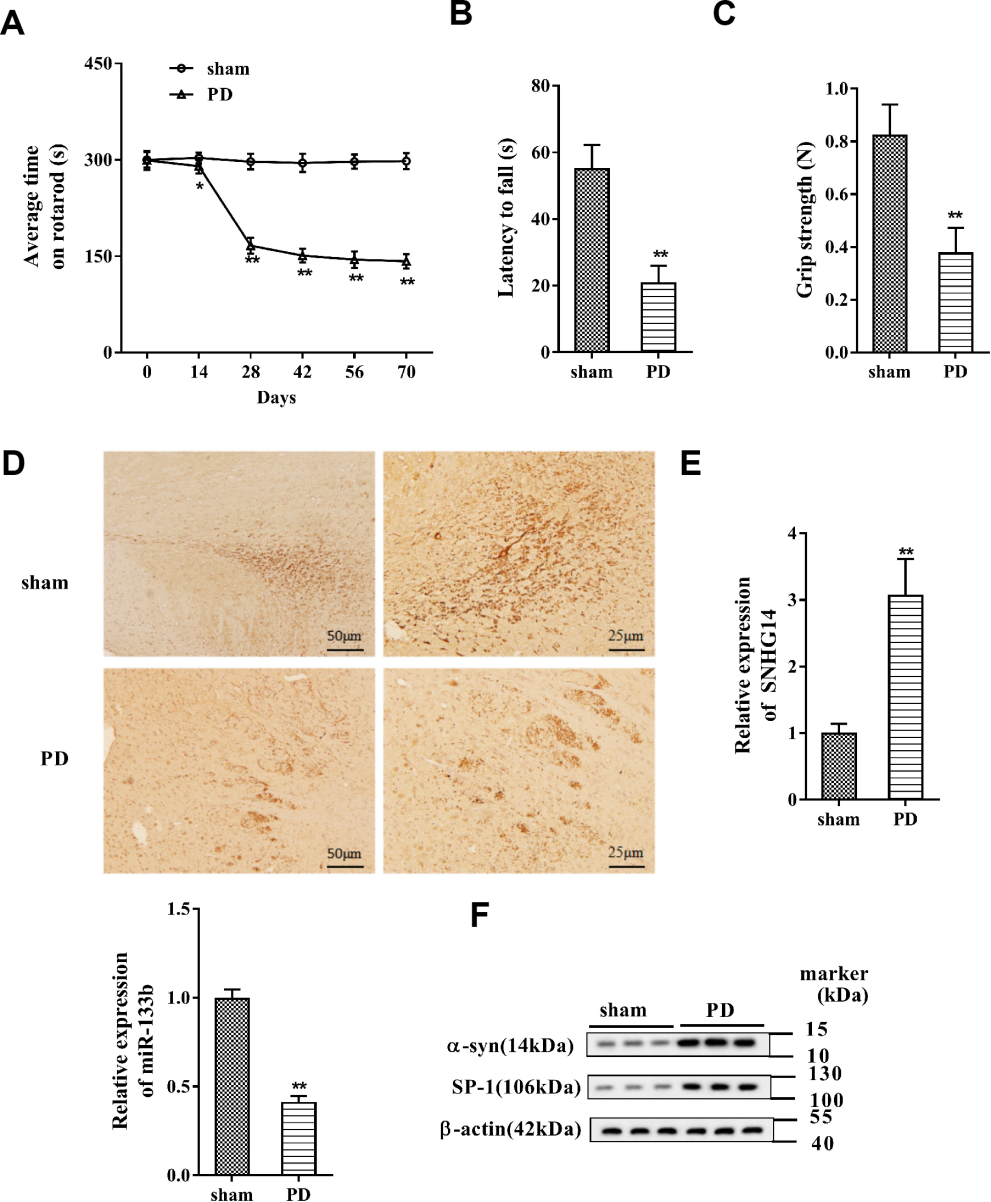
**SNHG14 expression was increased in PD mouse model.** PD mouse models (*n*=7) were constructed, with the sham group (*n*=7) served as control. The motor function of PD mice was assessed with (**A**) rotarod performance, (**B**) reversed screen test, and (**C**) forelimb grip strength test. (**D**) Immunohistochemistry staining was performed to determine the number of Tyrosine Hydroxylase (TH)-positive DA neuron in the brain tissues of mice. (**E**) Relative expression of SNHG14 and miR-133b in brain tissues of mice was quantified by qRT-PCR. (**F**) Protein level of α-syn and SP-1 in brain tissues of mice was analyzed with western blotting. *P<0.05, **P<0.01 compared with sham group.

PD mice were established as previously described [20], and the rotarod performance, inverted screen test, and forelimb grip strength test were performed. The results indicated that the average time on rotarod was dramatically shortened in PD mice (*n*=7) than that in the sham group (*n*=7) (Figure 1A), and the muscle strength of PD mice was significantly damaged (Figure 1B). In addition, the grip strength was clearly reduced in PD mice compared with that in the sham group (Figure 1C). In immunohistochemistry staining of brain tissues, the DA neuron was clearly reduced in PD mice, implying the significant DA neuron loss (Figure 1D). These data proved the pathological features of PD in the mouse model. In the brain tissue, the expression of SNHG14 was markedly elevated in PD mice when comparing to the sham group, while miR-133b was notably down-regulated (Figure 1E). What’s more, compared with the sham group, the α-syn and SP-1 protein levels were remarkably up-regulated in the brain tissue of PD mice (Figure 1F). These data showed that the expression of SNHG14 was elevated in both PD patients and PD mice.

### Binding between SP-1 and SNHG14 promoter was enhanced by rotenone

**Figure 2:**
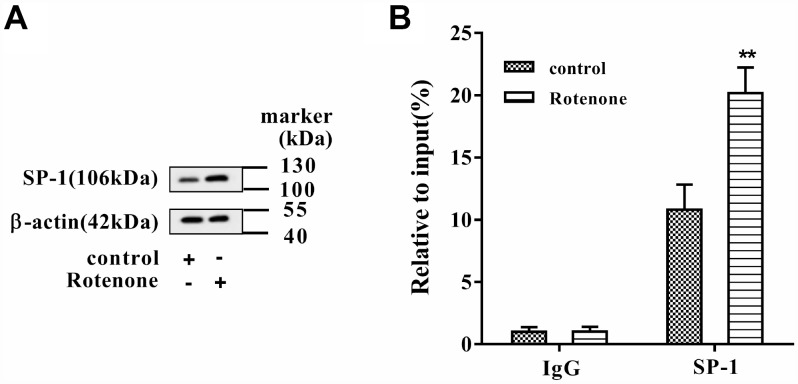
**The binding between SP-1 and SNHG14 promoter was influenced by rotenone.** Clonal mesencephalic DA cell line MN9D cells were treated with 1μmol/L rotenone. (**A**) SP-1 protein level in MN9D cells was determined by western blotting. (**B**) The binding activity of SP-1 to the promoter of SNHG14 was evaluated with chromatin immunoprecipitation (ChIP) assay. **P<0.01 compared with control.

After rotenone (1μmol/L) treatment in MN9D cells, we found that the level of SP-1 protein was increased (Figure 2A). Besides, the binding activity of SP-1 to the promoter of SNHG14 was augmented after rotenone treatment (Figure 2B). These results proved that rotenone promoted the binding between SP-1 and SNHG14 promoter.

### Rotenone promoted SNHG14 expression through SP-1

**Figure 3:**
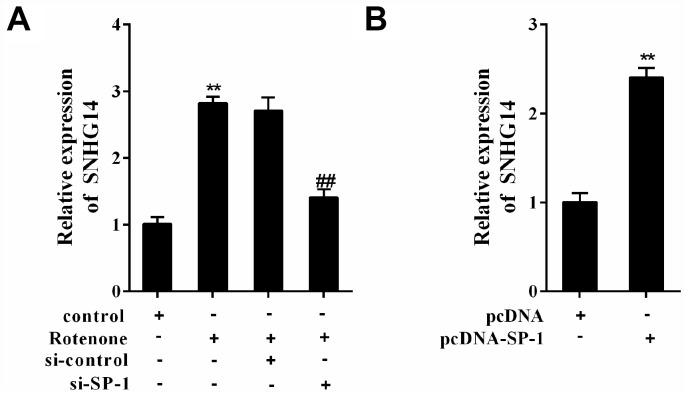
**SP-1 affected SNHG14 expression in rotenone-treated or -untreated MN9D cells.** MN9D cells were allocated into 4 groups: control, Rotenone (Ro, 1μmol/L), Ro+si-control, and Ro+si-SP-1, or allocated into 2 groups: pcDNA, pcDNA-SP-1. (**A** and **B**) Relative expression of SNHG14 was measured using qRT-PCR. **P<0.01 compared with control or pcDNA; ^##^P<0.01 compared with Ro+si-control.

MN9D cells were allocated into 4 groups: control, Rotenone (1μmol/L), Rotenone+si-control, and Rotenone +si-SP-1. It was shown that SNHG14 expression was facilitated by rotenone, but it was clearly suppressed with SP-1 interference (Figure 3A). As shown in Figure 3B, overexpression of SP-1 (with pcDNA-SP-1 transfected) increased SNHG14 expression when compared with its negative control, pcDNA. Here, we demonstrated that rotenone promoted SNHG14 expression through increasing the expression of SP-1.

### Interference of SNHG14 ameliorated DA neuron injury induced by rotenone

**Figure 4:**
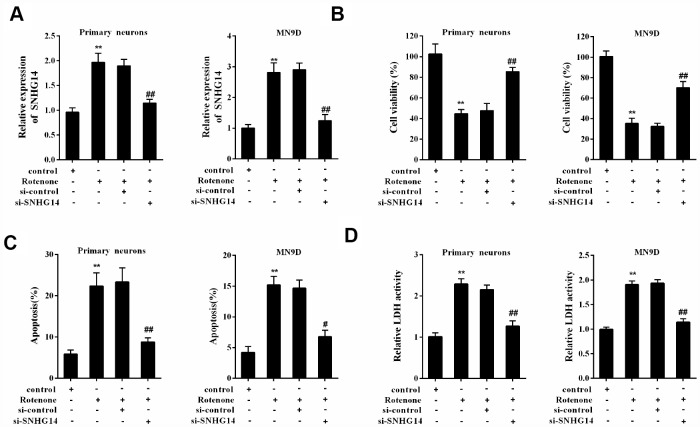
**Interference of SNHG14 ameliorated DA neuron injury induced by rotenone.** Primary mesencephalic neurons and MN9D cells were divided into 4 groups: control, Rotenone (Ro, 1μmol/L), Ro+si-control, and Ro+si-SNHG14. (**A**) The expression of SNHG14 in primary mesencephalic neurons and MN9D cells were measured by qRT-PCR. (**B**) Cells viability of two kinds of cells were detected by CCK8 assay. (**C**) Percentage of apoptotic cells in two kinds of cells was quantified using flow cytometry. (**D**) Relative LDH activity of two kinds of cells. **P<0.01 compared with control; ^##^P<0.01 compared with Ro+si-control.

Basing on si-SNHG14 transfection or rotenone (1μmol/L) treatment, primary mesencephalic neurons, which were isolated from mice, were divided into 4 groups: control, Rotenone, Rotenone+si-control, and Rotenone+si-SNHG14. The upregulation of SNHG14 expression was induced by rotenone in primary mesencephalic neurons, which was reversed with si-SNHG14 transfection (Figure 4A). Primary mesencephalic neurons activity was significantly inhibited by rotenone, but this inhibitory effect was reversed after si-SNHG14 transfection (Figure 4B). Numbers of apoptotic primary mesencephalic neurons induced by rotenone was reduced after si-SNHG14 transfection (Figure 4C). The LDH activity, which indicated the increase of cytotoxicity, was increased by rotenone but was inhibited by si-SNHG14 transfection in primary mesencephalic neurons (Figure 4D). In order to confirm the protective effect of si-SNHG14 in response to rotenone challenge more comprehensively, we replaced primary mesencephalic neurons with MN9D cells and repeated the above experiment. As shown in Figure 4A–4D, in MN9D cells, the stimulation of rotenone elevated the SNHG14 expression, decreased cell viability, induced cell apoptosis and increased cell cytotoxicity, while all these trends could be reversed by si-SNHG14 transfection. These findings revealed that interference of SNHG14 ameliorated neuron injury induced by rotenone.

### Interaction between SNHG14 and miR-133b in MN9D cells

**Figure 5:**
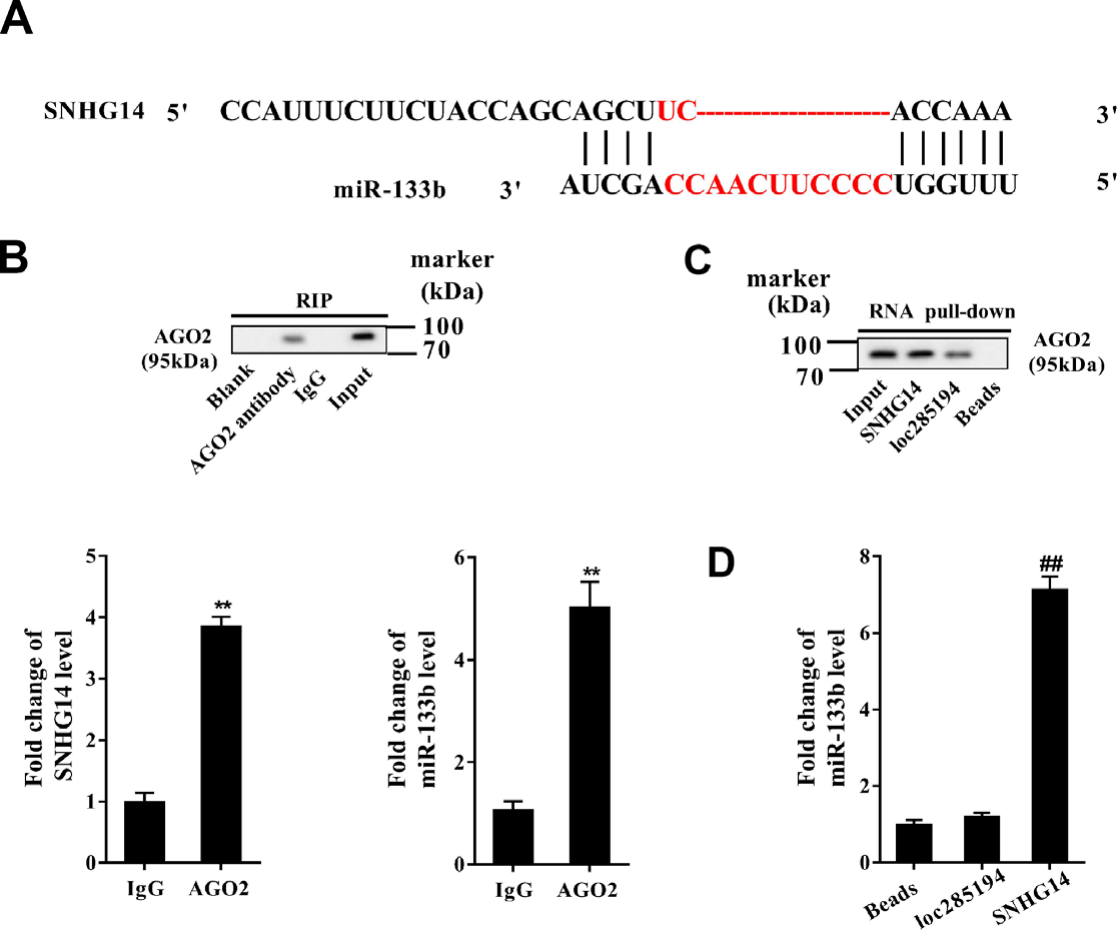
**Direct interactions between SNHG14 and miR-133b in MN9D cells.** (**A**) The potential binding sites between SNHG14 and miR-133b were shown. (**B**) AGO2-RNA immunoprecipitation (RIP) followed by qRT-PCR was conducted to determine SNHG14 endogenously associated with miR-133b. (**C**) RNA pull-down assay followed by western blotting determined AGO2 expression pulled down by biotin-labeled SNHG14. The use of biotin-labeled loc285194 was as a positive control for SNHG14. (**D**) The level of miR-133b in the pull-down complex was detected by qRT-PCR. **P<0.01 compared with IgG; ^##^P <0.01 compared with loc285194.

The interactional binding sites between SNHG14 and miR-133b were predicted via bioinformatics method (DIANA tools, Figure 5A), and the direct binding between of them in MN9D cells was evaluated with RIP and RNA pull-down assay. Compared with IgG, SNHG14 was abundantly detected in the AGO2 antibody precipitation complex (Figure 5B). In the RNA pull-down assay, plenty of AGO2 was found in the complex pulled down by biotin-labeled SNHG14 (Figure 5C). Furthermore, miR-133b was gathered in the complex pulled down by biotin-labeled SNHG14, comparing with in that of loc285194 (Figure 5D). These results identified miR-133b as a target of SNHG14.

### miR-133b negatively regulated α-syn expression

**Figure 6:**
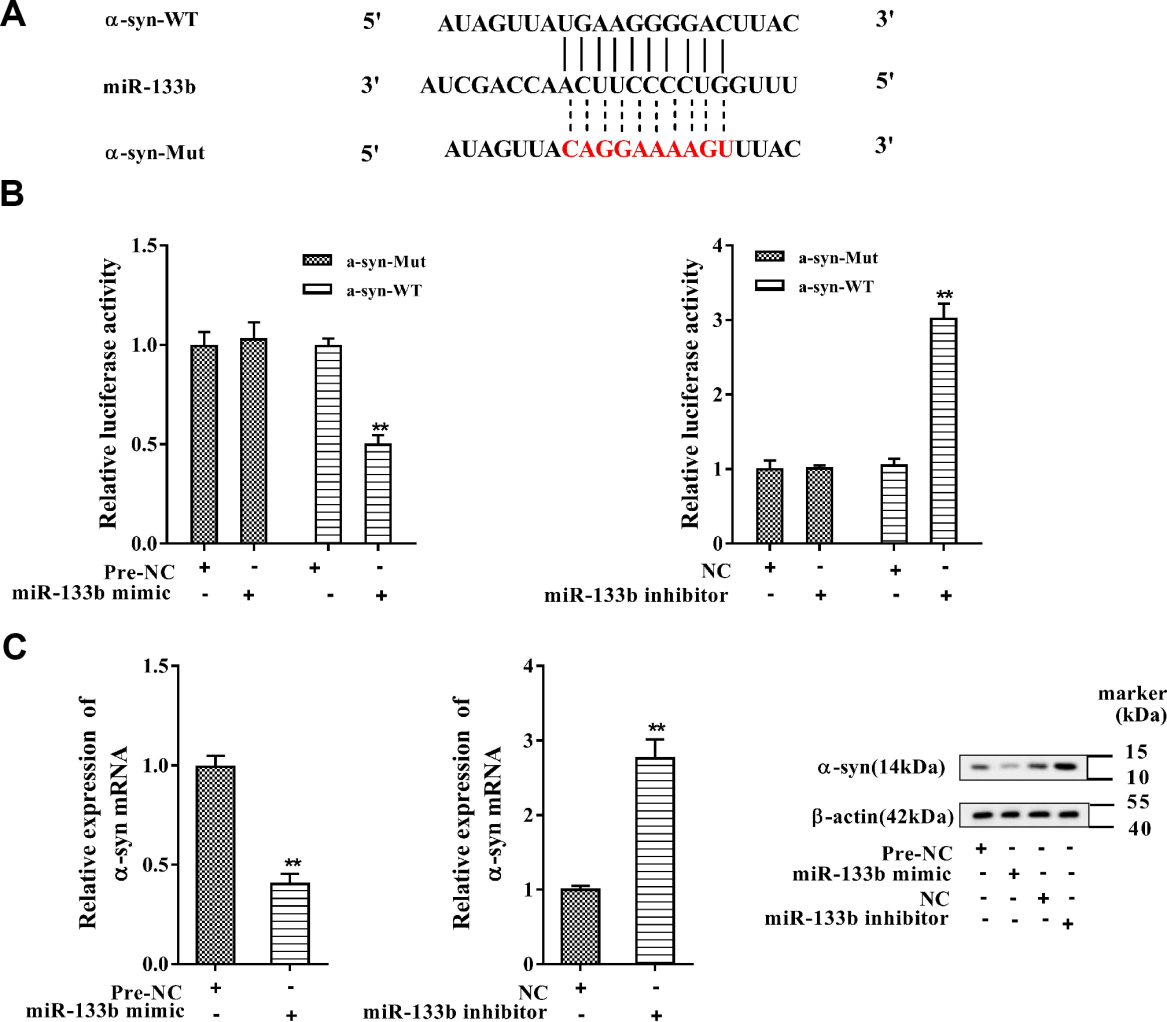
**MiR-133b negatively regulated α-syn expression.** (**A**) The complementary base pairs between miR-133b and α-syn 3′-UTR. (**B**) Regulatory role of miR-133b on α-syn expression was verified by using dual luciferase reporter gene assay. (**C**) The levels of α-syn mRNA and protein were analyzed with qRT-PCR and western blotting, respectively. **P<0.01 compared with pre-NC or NC.

In Figure 6A, the complementary base pairs between miR-133b and α-syn 3′-UTR were predicted by bioinformatics software (TargetScan), implying their potential interaction. With miR-133b mimic transfected into MN9D cells, the activity of α-syn wild type (WT) 3′-UTR was repressed when comparing to the negative control of miR-133b mimic (pre-NC); while miR-133b inhibitor transfection increased the activity when comparing to its negative control (NC) (Figure 6B). Instead, neither miR-133b mimic nor miR-133b inhibitor had influence on the activity of mutant (Mut) 3′-UTR of α-syn. With MN9D cells transfected with miR-133b mimic, the α-syn mRNA and protein levels were all dramatically dropped; but the expression of α-syn mRNA and protein in MN9D cells was markedly increased with miR-133b inhibitor transfected (Figure 6C). The data illuminated that miR-133b negatively modulated α-syn expression through targeting its 3′-UTR.

### SNHG14/miR-133b regulated α-syn expression

**Figure 7:**
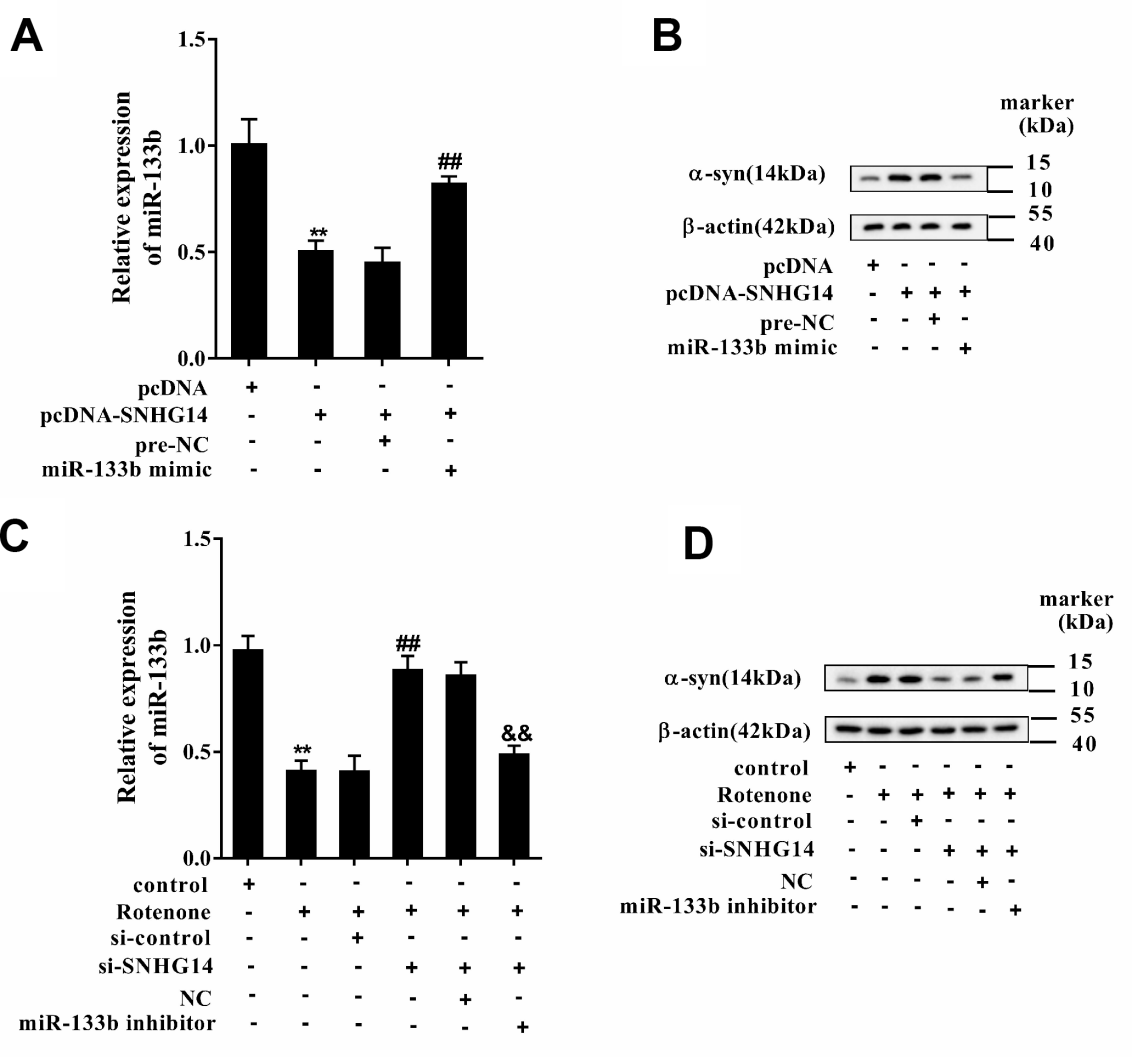
**SNHG14/miR-133b regulated α-syn expression. MN9D cells were transfected with various vectors and treated with or without rotenone.** (**A**) Relative expression of miR-133b was detected by qRT-PCR. (**B**) Level of α-syn protein was analyzed using western blotting. **P<0.01 compared with pcDNA; ^##^P<0.01 compared with pcDNA-SNHG14+pre-NC. (**C**) Relative expression of miR-133b was determined with qRT-PCR. (**D**) Level of α-syn protein was analyzed using western blotting. **P<0.01 compared with control; ^##^P<0.01 compared with Ro+si-control; ^&&^P<0.01 compared with Ro+si-SNHG14+NC.

Afterwards, MN9D cells were assigned into 4 groups: pcDNA, pcDNA-SNHG14, pcDNA-SNHG14+pre-NC, and pcDNA-SNHG14+miR-133b mimic. Compared with pcDNA, over-expression of SNHG14 after pcDNA- SNHG14 transfection decreased miR-133b level, which was reversed by miR-133b mimic (Figure 7A); while the alteration of α-syn level in each group was just on the opposite with that of miR-133b (Figure 7B). MN9D cells were then divided into another 6 groups: control, Rotenone, Ro+ si-control, Ro+si-SNHG14, Ro+si-SNHG14+NC, and Ro+si-SNHG14+miR-133b inhibitor. The results indicated that rotenone lowered miR-133b level, which was reversed with interference of SNHG14, but miR-133b expression was finally repressed by miR-133b inhibitor (Figure 7C). On the contrary, α-syn protein expression was elevated after rotenone treatment but reduced with SNHG14 interfered, which was terminally boosted by miR-133b inhibitor (Figure 7D). We demonstrated that SNHG14 regulated α-syn expression via miR-133b.

### Interference of SNHG14 improved DA neuron activity that affected by rotenone through miR-133b

**Figure 8:**
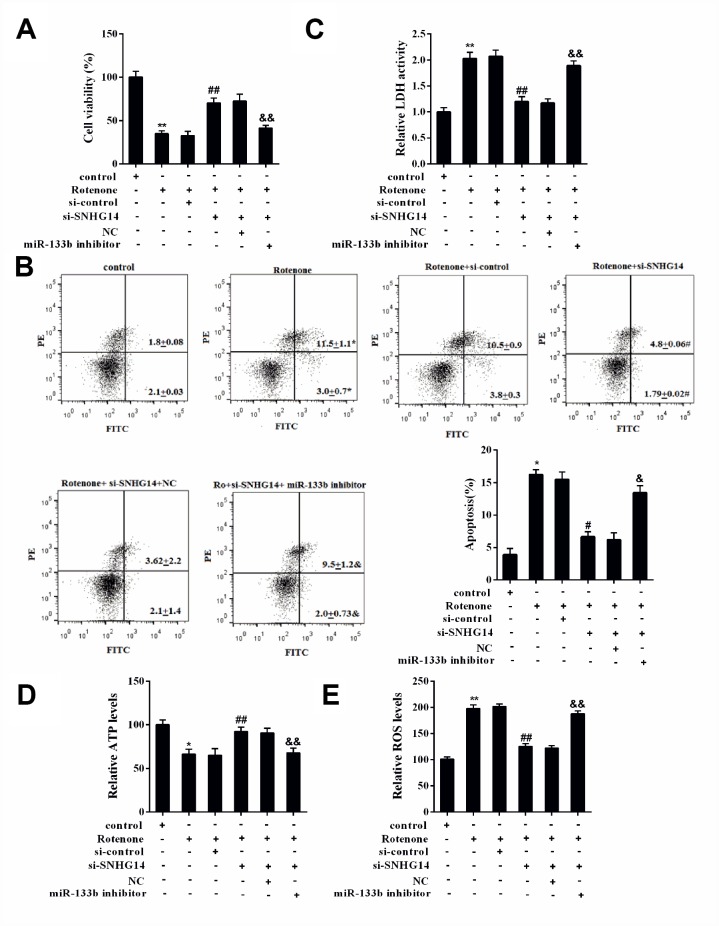
**SNHG14/miR-133b affected DA neuron activity that damaged by rotenone. MN9D cells were transfected with various vectors and treated with or without rotenone.** (**A**) Cells viability was detected by CCK8 assay. (**B**) Percentage of apoptotic cells was quantified using flow cytometry. (**C**) Relative LDH activity. (**D**) The level of ATP in each group was measured. (**E**) The level of ROS in each group was measured. **P<0.01 compared with control; ^##^P <0.01 compared with Ro+si-control; ^&&^P<0.01 compared with Ro+si-SNHG14+NC.

MN9D cells were divided into 6 groups: control, Rotenone, Rotenone+si-control, Rotenone+si-SNHG14, Rotenone+si-SNHG14+NC, and Rotenone+si-SNHG14+miR-133b inhibitor. Figure 8A displayed that cells activity was significantly inhibited by rotenone but enhanced after si-SNHG14 transfection, which was terminally repressed by miR-133b inhibitor. Neuron apoptosis (Figure 8B) and LDH activity (Figure 8C) were promoted by rotenone but suppressed by SNHG14 down-regulation, but they were both augmented by miR-133b inhibitor. Because the rotenone is an inhibitor of mitochondrial complex, to elaborate the effect of SNHG14/miR-133b pathway on mitochondrial function, we detected the level of ATP and ROS in rotenone–treated MN9D cells. As shown in Figure 8D–8E, the silencing of SNHG14 alleviated the rotenone-induced ATP synthesis reduction and ROS production increase, while miR-133b inhibitor reversed the effect of si-SNHG14. It could be concluded that interference of SNHG14 improved rotenone-damaged neuron activity through miR-133b.

### miR-133b alleviated neuron injury induced by rotenone via inhibiting α-syn expression

To prove whether α-syn was responsible for the protective effect of miR-133b on rotenone-damaged neuron cells, we overexpressed α-syn in MN9D cells, which had been transfected with miR-133b mimic before. As shown in Supplementary Figure 1C–1E, α-syn overexpression reversed the protective effect of miR-133b on cells, decreasing cell viability, inducing apoptosis, and enhancing rotenone-induced cytotoxicity. These data showed that α-syn played an important role in the protective effect of miR-133b on rotenone-induced neuron injury.

### Down-regulated SNHG14 mitigated neuron injury in PD mice

**Figure 9:**
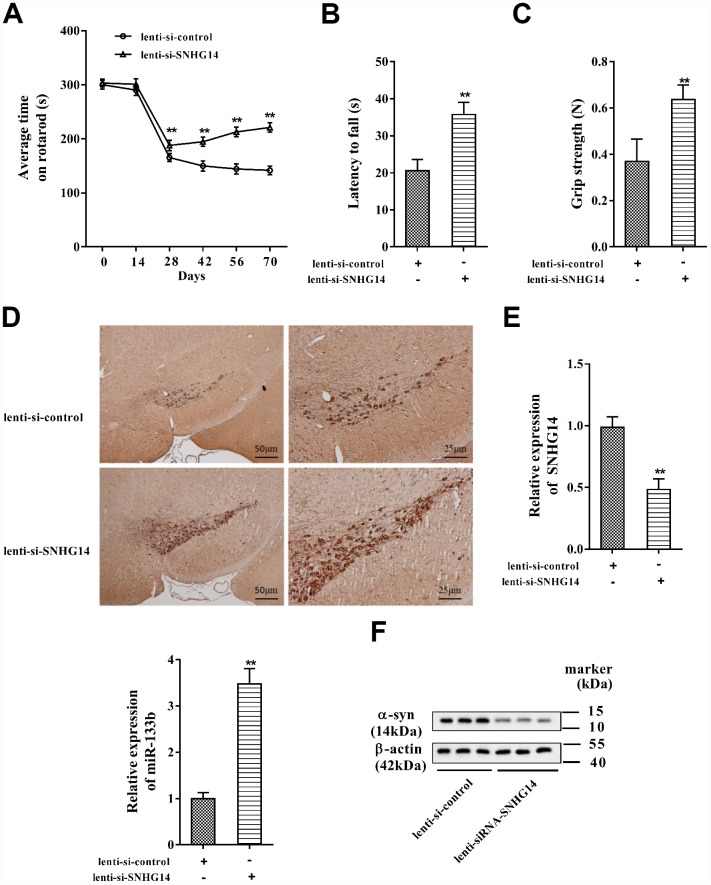
**Down-regulated SNHG14 mitigated neuron injury in PD mice.** PD mice (*n*=7) were injected with lenti-si-control or lenti-si-SNHG14. The motor function of mice was assessed with (**A**) rotarod performance, (**B**) reversed screen test, and (**C**) forelimb grip strength test. (**D**) Immunohistochemistry staining was performed to determine the number of TH-positive DA neuron in the brain tissues of mice. (**E**) Relative expression of SNHG14 and miR-133b in brain tissues of mice was evaluated with qRT-PCR. (**F**) Level of α-syn and SP-1 protein in brain tissues of mice was assessed by western blotting. **P<0.01 compared with lenti-si-control.

To validate the influence of SNHG14 interference on PD progression *in vivo*, PD mice (*n*=7 in each group) were injected with lenti-si-control or lenti-si-SNHG14, and the PD pathological characteristics were analyzed. As shown in Figure 9A, the average time on rotarod was dramatically prolonged in PD mice injected with lenti-si-SNHG14 than that in its control group, and the muscle strength of PD mice was notably improved (Figure 9B), as well as the grip strength (Figure 9C). The immunohistochemistry staining of brain tissues illustrated that the DA neuron loss in PD mice was remarkably improved after SNHG14 silence (Figure 9D). Meanwhile, lenti-si-SNHG14 injection effectively down-regulated the expression of SNHG14 in the brain tissue of PD mice, but it remarkably enhanced miR-133b expression (Figure 9E). Inversely, compared with the lenti-si-control group, the α-syn protein levels were clearly diminished in PD mice injected with lenti-si-SNHG14 (Figure 9F). Our findings verified that down-regulated SNHG14 mitigated neuron injury in PD mouse model.

## DISCUSSION

The etiology of PD have been explained by several hypotheses, apoptotic cell death and loss of dopaminergic neuron is considered one of the most prevalent mechanisms [21]. With PD mice model constructed and rotenone used to induce dopaminergic neuron damage in this study, we identified a regulatory pathway concerning PD-mediated dopaminergic neuron injury involving in SP-1, lncRNA SNHG14, miR-133b, and α-syn. Collectively, we concluded that low expression of SNHG14 mitigates dopaminergic neuron injury by down-regulating α-syn via miR-133b, which contributes to improving PD pathological state. With this study performed, the pathogenesis of PD was further understood; meanwhile, the significance of SNHG14 in PD and the neuroprotective effect of miR-133b were explained, providing novel perspectives into PD progression and treatment.

Accumulating evidences have illuminated that lncRNAs occupied essential places in diverse physiological and pathological processes and aberrant expression of them has been implicated in various complex disease [22]. As the second comment neurodegenerative disorder behind Alzheimer's disease, research on PD is widely paid attention, and the expression analysis of lncRNAs has emerged in recent years. Soreq et al. identified 13 low expressed lncRNAs in PD patients’ leukocytes through a whole-transcriptome RNA-Seq analysis, four of which were inversely altered post-Deep Brain Stimulation, providing a comprehensive new resource for understanding disease transcriptome modifications in PD [23]. In their study, lnc-FRG1-3 was thought to be related to the muscle rigidity in PD. Another lncRNA, post-DBS (RP11-79P5) was noted in PD brain RNA-Seq dataset and was claimed to be an exquisite biomarker for PD [24]. LncRNA MALAT1 has been confirmed to be associated with α-syn, leading to the increased stability and expression of α-syn in SH-SY5Y cells, and augmented α-syn expression in PD mice, exhibiting its crucial role in affecting PD progression [25]. In our study, the promoter of lncRNA SNHG14 binding by the transcription factor SP-1 was identified in DA neurons, and the binding between them was enhanced by rotenone therefore promoted SNHG14 expression. It was the first time that the action mechanism of rotenone was uncovered in PD development by regulating lncRNA level, and the involvement of SNHG14 in DA neuron injury was also highlighted.

So far, impact of SNHG14 on gastric cancer development has been discovered to constrain cell viability, migration, invasion, and promote cell apoptosis, by serving as a competing endogenous RNA of miR-145 [26]. In cerebral infarction and glioma, the function of SNHG14 has also been reported [19, 27], suggesting its association with neurological disorders. In the present study, we indicated the up-regulation of SNHG14 in PD patients, PD model mice and in DA neuron treated with rotenone, and manifested that SNHG14 was transcriptionally activated by SP-1 in DA neurons. By loss-of-function analysis, we illuminated that interference of SNHG14 alleviated DA neuron injury induced by rotenone and in PD mice. This study emphasized the function of SNHG14 in participating in DA neuron injury and PD progression, exhibiting its promising role as an effective target in PD treatment.

Our results suggested that miR-133b was firstly identified as a novel target of SNHG14, hinting that the function of SNHG14 may be uncovered in more human diseases related to miR-133b. Apart from its role in PD, miR-133b served as a cancer suppressor by repressing proliferation, migration, invasion and cell cycle in gastric cancer and bladder cancer [28, 29]. On the other hand, miR-133b was dispensable for development, survival and regeneration of skeletal muscle [30], suggesting that muscle rigidity in PD may attribute to down-regulation of miR-133b, which deserves further study. Our study illustrated the down-regulation of miR-133b in dopaminergic neuron injury induced by rotenone, as well as its neuroprotective role in PD by targeting α-syn. This study was in accordance with the previous study holding the view that reduced circulating levels of miR-133b showed its potential role as a biomarker for PD [31]. The current study elucidated a SNHG14/miR-133b/α-syn signaling pathway in DA neurons, and clarified the regulatory effect of rotenone on SNHG14 expression via SP-1, and these findings may provide new perspective for PD prevention, diagnosis, and therapy.

The α-syn protein deposition is well recognized to contribute to the pathogenesis of PD, which may exert deleterious effects on neighboring cells, including seeding of aggregation, or affecting the number of DA neurons, thus possibly facilitating disease propagation [32, 33]. This study not only identified miR-133b as a new regulator of α-syn, but also disclosed regulatory effect of SNHG14 on α-syn through endogenously competing with miR-133b. With strategies based on targeting α-syn developed [34], we believe that gratifying progress may be achieved on the treatment of PD.

In conclusion, we clarified that interference of lncRNA SNHG14 expression mitigates dopaminergic neuron injury by down-regulating α-syn via miR-133b, which contributes to improving PD pathological state. Our study highlighted the role of SNHG14 in PD pathogenesis, and emphasized its regulatory relationship with SP-1 and miR-133b, providing significant theoretical foundation for developing novel therapies for PD and other neurodegenerative disorders.

## MATERIALS AND METHODS

### PD mice model

PD mice were established as previously described [20]. C57BL/6 mice (7-week-old) were purchased from the laboratory animal center of Zhengzhou University, and kept under a 12h light/dark cycle at room temperature with free access to food and water. To establish PD mice model, mice were anesthetized with isoflurane (Sigma, MO, USA) and underwent a stereotaxic surgery. A hole was drilled in the skull, then a cannula was inserted in the right striatum. Freshly prepared rotenone (5.4μg rotenone dissolved in 2μl DMSO; Sigma, MO, USA) was infused through the cannula. Stereotaxic coordinates were used as the follows: AP+0.4, ML-2.0 (from bregma), and DV-3.3 (below dura). In sham group, mice were injected with DMSO, and the other procedures were similar as PD mice. The final concentration of DMSO injected into the substantia nigra of mice in this study was 1% (diluted with 0.9% normal saline). All animal experiments were approved by the Ethics Committee of the First Affiliated Hospital of Zhengzhou University.

### Motor function assessment

Rotarod test was used to assess the motor function of the mice. Mice were placed on an accelerating rod. The speed was started at 2 rpm and gradually increased to 20 rpm. Time to first fall of the mice was recorded for a maximum of 300 s. The test was performed every 14 days until day 70.

Muscle strength of the four limbs and muscular forelimb strength were measured by latency to fall and grip strength on day 70 after surgery using the inverted screen test and forelimb grip strength test. The mouse was placed in the center of a wire mesh screen, which was subsequently rotated to an inverted position. Latency to fall was measured in seconds. Muscular forelimb strength was detected by a grip strength tester.

### Immunohistochemistry

Immunohistochemistry staining was performed to determine the level of Tyrosine Hydroxylase (TH) in the brain tissues of mice. Coronal slices were sectioned and incubated with 0.3% H_2_O_2_ for 30min. After blocking with goat serum, the rabbit anti-TH (1: 1500) was added for incubation overnight, and the sections were washed with PBS. Biotinylated secondary antibody (1:500) was added to incubate for 2h and then the color was developed with 3,3′-diaminobenzidine tetrachloride (DAB).

### Isolation and culture of primary mesencephalic neurons

Primary mesencephalic neurons were isolated as previously described [35]. In brief, the midbrain was dissected from newborn mice pups. Then the tissues were chemically and mechanically dissociated into single cell suspensions. Cells were resuspended in Dulbecco’s modified Eagle medium (DMEM; ThermoFisher Scientific, CA, USA) containing 10% FBS (ThermoFisher Scientific, CA, USA) and plated into polyD-lysine coated 6-well plates at a density of 1×10^5^ per well. Six hours after plating, the medium was replaced with fresh Neurobasal medium (ThermoFisher Scientific, CA, USA) supplemented with B27 supplement (Invitrogen, CA, USA), and incubated for a further 5 days.

### Cell culture and transfection

Murine dopaminergic cell line MN9D was purchased from the American Type Culture Collection (USA), and cultured in DMEM/ F-12 (ThermoFisher Scientific, CA, USA) supplemented with 10% fetal bovine serum (FBS; ThermoFisher Scientific, CA, USA), 100 U/ml penicillin (Sigma, MO, USA), and 100 μg/ml streptomycin (Sigma, MO, USA) at 37°C with 5% CO_2_.

Recombinant plasmid pcDNA-SP-1 was constructed by inserting SP-1 cDNA into pcDNA3.1 (ThermoFisher Scientific, CA, USA). Plasmid pcDNA-SNHG14 was constructed by inserting SNHG14 cDNA into pcDNA3.1 (ThermoFisher Scientific, CA, USA). For SP-1 knockdown, small interference RNA targeting SP-1 (si-SP-1) was synthesized by Ribobio (Guangzhou, China). For SNHG14 knockdown, small interference RNA targeting SNHG14 (si-SNHG14) was synthesized by Ribobio (Guangzhou, China). MN9D cells were seeded at 4×10^5^ cells/well in a 6-well plate and cultured until 70% confluence, then transfected with 50nmol si-SP-1, pcDNA-SP-1, si-SNHG14, pcDNA-SNHG14, miR-133b mimic, miR-133b inhibitor and their corresponding negative controls using transfection reagent (ThermoFisher Scientific, CA, USA).

### Lentiviral injection

For observation the effect of SNHG14 knockdown on PD mice, lentiviral vector lenti-si-SNHG14 or lenti-si-control (Ribobio, Guangzhou, China) was injected into the right substantia nigra pars compacta SNpc (AP: -2.7, ML: +1.0, DV: -4.5) [36]. According to the previous report [37], this procedure was conducted using a 5-μl Hamilton syringe with a 33-gauge tip needle, with 1 μl/side at a rate of 0.2μl/min for 10 min. Two days later, the PD mice model was established as above described, and the mice were divided into lenti-si-control (*n*=7) and lenti-si-SNHG14 group (*n*=7).

### Quantitative real-time PCR (qRT-PCR)

Total RNAs were extracted from brain tissues or MN9D cells or primary mesencephalic neurons using Trizol reagent (ThermoFisher Scientific, CA, USA), and inversely transcribed into cDNA using the High Capacity cDNA Reverse Transcription Kit (ThermoFisher Scientific, CA, USA). Quantitative real-time PCR was performed to measure SNHG14, miR-133b and α-syn expressions using SuperScript III Platinum SYBR Green One-Step qRT-PCR Kit (ThermoFisher Scientific, CA, USA). The relative expression of SNHG14, miR-133b and α-syn were expressed as a function of threshold cycle (Ct) and analyzed by 2^-ΔΔCt^ method.

### Western blotting

The proteins of brain tissues or MN9D cells were extracted using RIPA buffer (ThermoFisher Scientific, CA, USA). BCA assay (Beyotime Biotechnology, Nantong, China) was used to quantify protein concentrations. After boiling for 5 min at 95°C, the protein samples (50 μg) were separated using sodium dodecyl sulphate-polyacrylamide gel electrophoresis (SDS-PAGE) and transferred to polyvinylidene difluoride membranes (PVDF; ThermoFisher Scientific, CA, USA). The membranes were blocked with 5% skim milk for 1 h at room temperature. Then, the membranes were incubated with the primary antibodies against α-syn (ThermoFisher Scientific, CA, USA), SP-1 (ThermoFisher Scientific, CA, USA) or β-actin (Abcam, MA, USA) at 4°C overnight. The membranes were then incubated with the horseradish peroxidase-conjugated secondary antibodies (Abcam, MA, USA) for 2 h at room temperature. The blotted proteins were visualized using the ECL Western blotting substrate (ThermoFisher Scientific, CA, USA) and the intensity of band was quantified using image software (Bio-Rad, CA, USA).

### Measurement of ATP

Cells were incubated for 15 min with buffer containing 5 mM glucose alone or in combination with 100 μM glutamate or 10 mM azide or 1 mM iodoacetate + 10 mM azide. After centrifugation, protein content was assayed and ATP content was measured using ATP Determination Kit (Molecular Probes, Eugene, OR) according to the manufacturer's instructions. The amount of ATP within the samples was calculated from ATP standard curves.

### ROS staining and detection

Cells were collected and re-suspended in PBS at the concentration of 1×10^5^ cells/mL, then incubated with DCFH-DA (Nanjing institute of biological engineering, China) for 45 min at 37 °C, untreated cells served as negative control. ROS generation was analyzed using flow cytometer (BD Biosciences, San Jose, CA)

### Lactate dehydrogenase (LDH) cytotoxicity assay

The LDH release assay was performed to evaluate the cytotoxicity of rotenone-induced MN9D cells or rotenone-induced primary neurons. Briefly, cells in 96-well plates were measured using a LDH-Cytotoxicity Assay Kit (Abcam, Camb, UK) according to the manufacture’s protocols, and the absorbance of formazan in each well was read at a wavelength of 500nm on a microplate reader (ThermoFisher Scientific, CA, USA).

### Chromatin immunoprecipitation (ChIP) assay

ChIP assay was conducted using Pierce Magnetic ChIP Kit (ThermoFisher Scientific, CA, USA) according to the manufacturer’s instruction. Anti-SP-1 antibody was used to precipitate the DNA-protein complex. qRT-PCR was performed to detect the immunoprecipitated DNA.

### Cell viability analysis

Cell viability was detected by the Cell Counting kit-8 (CCK-8; Sigma, MO, USA) according to the manufacturer’s instruction. MN9D cells or primary neurons were seeded in 96-well plates and cultured overnight. The cells were transfected with si-control, si-SNHG14, negative control, or miR-133b inhibitor, then treated with 1 μmol/l rotenone (Sigma, MO, USA) in 100 μl DMEM/F-12 medium (ThermoFisher Scientific, CA, USA) supplemented with 10% FBS (ThermoFisher Scientific, CA, USA) for 24 h. CCK-8 solution (100 μl/well) was added to each well after 24 h and incubated for 4 h at 37°C. The absorbance of each well was measured at 450 nm using a microplate reader (Bio-Rad, CA, USA).

### RNA immunoprecipitation (RIP) and RNA pull-down

RIP assay was performed using EZ-Magna RIP RNA-Binding Protein Immunoprecipitation Kit (Merck Millipore, MO, USA) according to the manufacturer’s instruction. MN9D cell lysate was prepared by 1.5×10^7^ cells using 100 μl RIP lysis buffer containing 0.25 μl RNase inhibitor and 0.5 μl protease inhibitor. The lysate was incubated with protein A/G magnetic beads conjugated to AGO2 antibody or normal mouse IgG. Subsequently, RNA-binding protein complex was obtained. IP-western was used to assess AGO2 protein level, and qRT-PCR was used to detect lncRNA SNHG14 and miR-133b.

The interaction between SNHG14 and miR-133b was further detected by RNA pull-down assay. The biotin labeled SNHG14 was transcribed with the Biotin RNA Labeling Mix (Roche, Basel, Switzerland) and T7 RNA polymerase (Roche, Basel, Switzerland). MN9D cell lysate was prepared by 1.5×10^7^ cells in 100 μl RIP lysis buffer, and mixed with 50 pmol biotin-labeled SNHG14 and 50 μl streptavidin agarose magnetic beads for 1 h at 4°C. Then, RNA-binding protein complex was boiled in SDS buffer for 10 min. AGO2 protein level was measured by western blot, and miR-133b in SNHG14 pull-down complex was detected by qRT-PCR using loc285194 as positive control.

### Dual luciferase reporter gene assay

The interaction between miR-133b and the α-syn 3′UTR was confirmed by dual-luciferase reporter gene assay. Based on the putative miR-133b binding sites in the α-syn 3′UTR (α-syn-WT), the α-syn 3′UTR with mutated miR-133b binding sites (α-syn-Mut) was generated. Luciferase reporter vectors containing α-syn-WT (pGL-α-syn-WT) or α-syn-Mut (pGL-α-syn-Mut) was constructed by Ribobio (Guangzhou, China). pGL-α-syn-WT or pGL-α-syn-Mut combined with miR-133b mimic or miR-133b inhibitor were co-transfected into MN9D cells using transfection reagent (ThermoFisher Scientific, CA, USA). After 48 hours of the transfection, luciferase activity was measured by Dual Luciferase Assay System (Promega, WI, USA) according to the manufacturer’s instructions.

### Statistical analysis

Experimental results were expressed as mean±standard deviation (SD) and analyzed using SPSS (version 22.0, USA). The differences were analyzed by student t test or one-way analysis of variance (ANOVA) with the Newman-Keuls post hoc test. Results were considered statistically significant when p<0.05.

## Supplementary Material

Supplementary Figure 1
