# Supplementary material for: Dopaminergic neuron injury in Parkinson’s disease is mitigated by interfering lncRNA SNHG14 expression to regulate the miR-133b/ α-synuclein pathway
Source: Aging (Albany NY). 2019 Nov 4;11(21):9264–79. doi: 10.18632/aging.102330 (PMC6874444; doi:10.18632/aging.102330)
Supplement: Supplementary Figure 1 [file aging-11-102330-s001.pdf]

SUPPLEMENTARY FIGURE

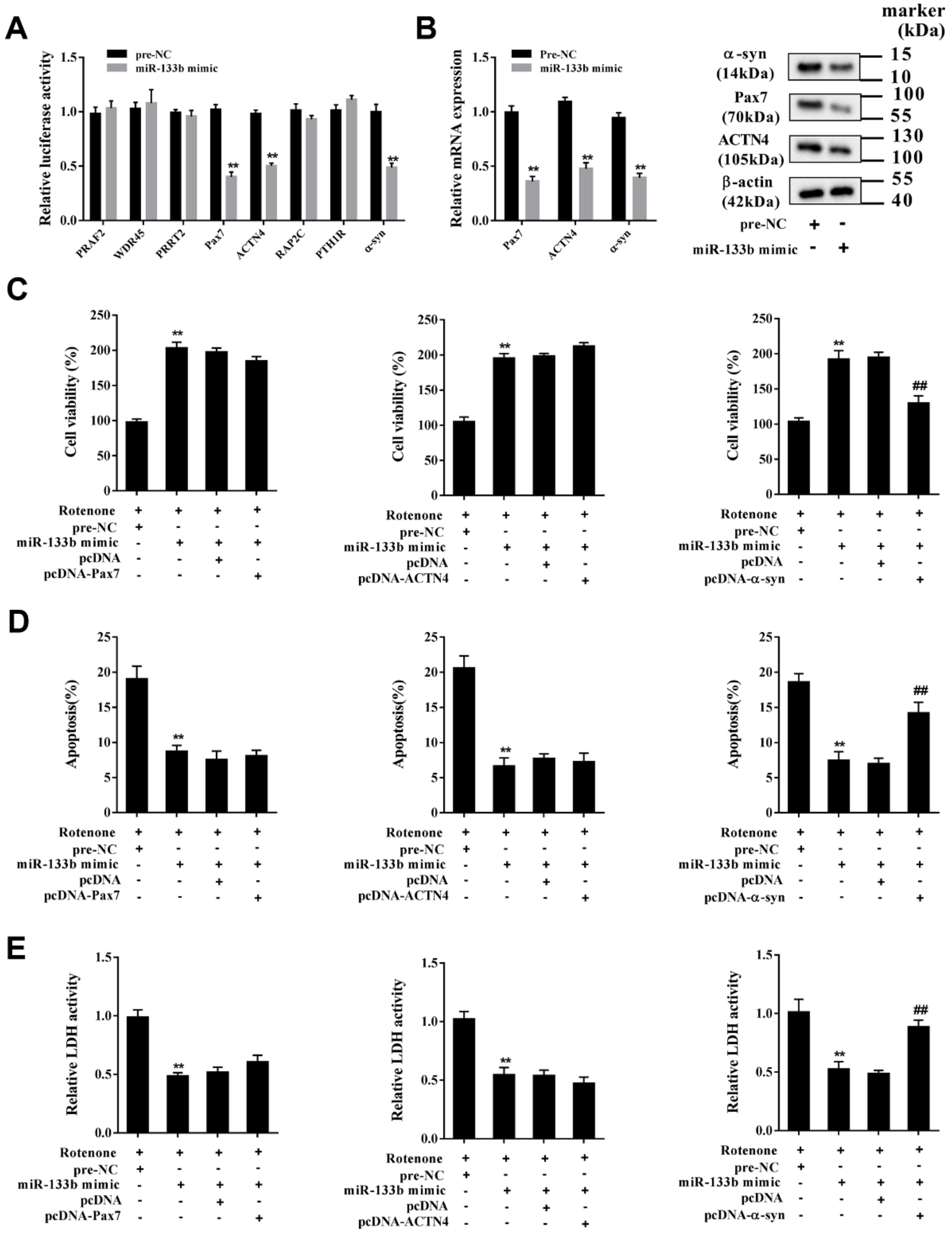

Supplementary Figure 1. (**A**) The 3'UTR activity of several target genes of miR-133b was measured using dual luciferase reporter gene assay. (**B**) The expression levels of Pax7, ACTN4 and α-syn were measured using qRT-PCR and western blotting. Pax7, ACTN4 and α-syn was overexpressed respectively in MN9D cells, which had been transfected with miR-133b mimic. (**C**) Cell viability, (**D**) percentage of apoptosis, (**E**) LDH activity of each group was measured after rotenone treatment (1μmol/L). **P<0.01 compared with pre-NC, $^{\#\#}$P <0.01 compared with pcDNA.
